# Expert suggestion for diabetes management during the recent COVID‐19 pandemic

**DOI:** 10.1111/1753-0407.13293

**Published:** 2022-07-17

**Authors:** Weiqing Wang, Jianmin Liu, Yifei Zhang, Guang Ning

**Affiliations:** ^1^ Department of Endocrine and Metabolic Diseases, Shanghai National Clinical Research Center for Metabolic Diseases, Ruijin Hospital Shanghai Jiao Tong University School of Medicine Shanghai China

Currently, a new wave of coronavirus disease 2019 (COVID‐19) pandemic appears worldwide with the omicron variant dominating. Compared to the other variants of SARS‐CoV‐2, omicron is more transmissible and with a shorter incubation period than the other variants such as delta, albeit with a reduced impact on lower respiratory tract and rates of hospital admission.^1^, ^2^

Acute and chronic infections are the common complications of diabetes. Although there is insufficient evidence to confirm that patients with diabetes are more susceptible to COVID‐19 than the general population, multiple studies have shown that the patients' underlying condition such as diabetes was associated with an increased risk of poorer clinical outcomes including mortality for these patients.^3^, ^4^, ^5^, ^6^, ^7^ Moreover, patients' metabolic control status including the glycemic control target achievement before infection and during hospitalization due to COVID‐19 both have an important impact on the natural course of this disease.^7^

Here, we propose this expert suggestion to provide necessary recommendation for the comprehensive multifactorial management of diabetes patients during the current COVID‐19 pandemic, especially under the current omicron wave.

## COVID‐19 PREVENTION

1. For patients with well‐controlled diabetes, COVID‐19 vaccines should be given in time. Moreover, after evaluation, booster vaccination should be encouraged for those who are eligible.
2. To reduce gathering, maintain social distance, wear masks, quit smoking, reduce alcohol consumption, balance nutrition, and exercise properly are key tips of lifestyle intervention for COVID‐19 prevention.
3. Active weight control with a target body mass index (BMI) < 25 kg/m^2^ is proper for most diabetes patients.
4. Comprehensive management of blood glucose, blood pressure, and lipid levels according to the principles of hierarchical management should be achieved.^8^, ^9^, ^10^
  (1) Glycated hemoglobin (HbA1c) control target: In general, HbA1c control target is <7%. Appropriate adjustment in combination with patient's age, diabetes duration, comorbidities, risk of hypoglycemia, and expected life span is necessary.
  (2) For diabetes patients with hypertension, the blood pressure control target is generally <130/80 mm Hg.
  (3) Low‐density lipoprotein cholesterol should be controlled below 2.6 mmol/L (100 mg/dl) for diabetes patients and further adjustment according to the patient's atherosclerotic cardiovascular disease risk is appropriate.
  (4) Try to use medications with sufficient clinical evidence including metformin, glucagon‐like peptide‐1 receptor agonists and sodium‐glucose cotransporter 2 inhibitors for glycemic control, statins for lipid lowering, and renin‐angiotensin system inhibitors for blood pressure control, so as to reduce the risks for cardiovascular disease comorbidities, diabetic microvascular complications, and all‐cause mortality in type 2 diabetes patients.

## GENERAL TREATMENT FOR COVID‐19

Adequate rest and nutritional support should be strengthened; vital signs, biochemical parameters, and chest imaging should be monitored; at the same time, antiviral, supportive treatment and other comprehensive management could be performed. Details can be found in the “Diagnosis and treatment plan for COVID‐19 (trial version 9)”.^1^

## METABOLIC CONTROL FOR DIABETES PATIENTS WITH COVID‐19

1 Basic requirements
  (1) Ensuring adequate sleep, setting up a regular routine, and self‐monitoring of blood glucose are all necessary.
  (2) Reduce the intake of foods with high glycemic index, such as starches, high‐sugar fruits, sugar‐sweetened beverages, etc., and appropriately increase the intake of high‐quality protein.
  (3) Taking moderate or low‐intensity exercise, about 150 min a week, divided into 4‐5 times to accomplish the sports. Selecting an appropriate exercise mode according to the patient's physical condition and range of motion are necessary. Marching on the spot, tai chi, and calisthenics are optional indoor sports during the pandemic.
2 Target of glycemic control and medication selection for asymptomatic/mild symptoms patients
  (1) Patients aged <65 years and without obvious chronic complications of diabetes: maintaining fasting blood glucose at 6.1–7.8 mmol/L and 2‐h postprandial blood glucose: 7.8–10.0 mmol/L is recommended.
  (2) Patients aged ≥65 years, or with serious diabetes related comorbidities, a less stringent fasting blood glucose at 7.8–10.0 mmol/L, 2‐h postprandial blood glucose at 7.8–12.0 mmol/L is recommended.
  (3) Previous medications can be maintained for those with stable glycemic control status. If blood glucose concentration increases continuously, switching to insulin administration is preferred.
  (4) If hypoglycemic symptoms (such as palpitations, trembling, sweating, hunger, etc.) occur, fingerstick blood glucose should be taken immediately, and sugary foods should be taken as soon as possible, such as juice, glucose water, candy, etc. If the symptoms cannot be relieved or aggravated, an intravenous bolus of glucose should be given quickly. At the same time, discontinuing current hypoglycemic agents and monitoring blood glucose are necessary. Hypoglycemic medication should be adjusted subsequently.
3 For patients hospitalized with common or severe COVID‐19: stratified management of blood glucose and subcutaneous insulin administration or intravenous insulin injection for certain conditions is recommended.^10^, ^11^

### Telemedicine application during pandemic

With the continuous development of Internet of things technology, telemedicine has been applied widely. Therefore, during the COVID‐19 pandemic, the use of Internet‐based platforms outside of hospitals including specialized medical apps or certain social software are all efficient supplements for remote consultation and monitoring for sustained metabolic control and medication adjustment for these patients, as well as reducing the potential risks associated with hospital visits.^12^

## CONFLICT OF INTEREST

None declared.

## References

[1] National Health Commission of the People's Republic of China . Diagnosis and treatment plan for COVID‐19 (trial version 9). Article in Chinese. Inter J Epidemiol Infect Dis. 2022;49(2):73‐80. doi:10.3760/cma.j.cn331340-20220325-00065

[2] Zhang X , Zhang W , Chen S . Shanghai's life‐saving efforts against the current omicron wave of the COVID‐19 pandemic. Lancet. 2022;399(10340):2011‐2012. doi:10.1016/s0140-6736(22)00838-8 35533708 PMC9075855

[3] Seiglie J , Platt J , Cromer SJ , et al. Diabetes as a risk factor for poor early outcomes in patients hospitalized with COVID‐19. Diabetes Care. 2020;43(12):2938‐2944. doi:10.2337/dc20-1506 32847827 PMC7770271

[4] Barron E , Bakhai C , Kar P , et al. Associations of type 1 and type 2 diabetes with COVID‐19‐related mortality in England: a whole‐population study. Lancet Diabetes Endocrinol. 2020;8(10):813‐822. doi:10.1016/s2213-8587(20)30272-2 32798472 PMC7426088

[5] Hartmann‐Boyce J , Rees K , Perring JC , et al. Risks of and from SARS‐CoV‐2 infection and COVID‐19 in people with diabetes: a systematic review of reviews. Diabetes Care. 2021;44(12):2790‐2811. doi:10.2337/dc21-0930 34711637 PMC8669527

[6] Menni C , Valdes AM , Polidori L , et al. Symptom prevalence, duration, and risk of hospital admission in individuals infected with SARS‐CoV‐2 during periods of omicron and delta variant dominance: a prospective observational study from the ZOE COVID study. Lancet. 2022;399(10335):1618‐1624. doi:10.1016/s0140-6736(22)00327-0 35397851 PMC8989396

[7] Yonekawa A , Shimono N . Clinical significance of COVID‐19 and diabetes: in the pandemic situation of SARS‐CoV‐2 variants including omicron (B.1.1.529). Biology. 2022;11(3):400. doi:10.3390/biology11030400 35336774 PMC8945151

[8] Chan JCN , Lim L‐L , Wareham NJ , et al. The lancet commission on diabetes: using data to transform diabetes care and patient lives. Lancet. 2020;396(10267):2019‐2082. doi:10.1016/s0140-6736(20)32374-6 33189186

[9] Chinese Diabetes Society . Guideline for the prevention and treatment of type 2 diabetes mellitus in China(2020 edition). Article in Chinese. Chin J Endocrinol Metab. 2021;37(4):311‐398. doi:10.3760/cma.j.cn311282-20210304-00142

[10] Wang W , Lu J , Gu W , Zhang Y , Liu J , Ning G . Care for diabetes with COVID‐19: advice from China. J Diabetes. 2020;12(5):417‐419. doi:10.1111/1753-0407.13036 32285556

[11] Chinese Endocrinologist, Chinese Medical Doctor Association, Expert group of blood glucose management for Chinese inpatients . Expert consensus on blood glucose management for Chinese inpatients. Chin J Endocrinol Metab. 2017;33(1):1‐10. doi:10.3760/cma.j.issn.1000-6699.2017.01.001

[12] Zhang Y , Wang W , Ning G . Metabolic management center: an innovation project for the management of metabolic diseases and complications in China. J Diabetes. 2019;11(1):11‐13. doi:10.1111/1753-0407.12847 30284373

